# Genetic background influences tumour development in heterozygous *Men1* knockout mice

**DOI:** 10.1530/EC-20-0103

**Published:** 2020-04-28

**Authors:** Kate E Lines, Mahsa Javid, Anita A C Reed, Gerard V Walls, Mark Stevenson, Michelle Simon, Kreepa G Kooblall, Sian E Piret, Paul T Christie, Paul J Newey, Ann-Marie Mallon, Rajesh V Thakker

**Affiliations:** 1 Academic Endocrine Unit, Radcliffe Department of Medicine, University of Oxford, Oxford Centre for Diabetes, Endocrinology and Metabolism (OCDEM), Churchill Hospital, Headington, Oxford, UK; 2 MRC Harwell Institute, Mammalian Genetics Unit, Harwell Campus, Oxfordshire, UK

**Keywords:** genetic modifiers, pancreatic neuroendocrine tumour, mouse strain, menin, pituitary

## Abstract

Multiple endocrine neoplasia type 1 (MEN1), an autosomal dominant disorder caused by *MEN1* germline mutations, is characterised by parathyroid, pancreatic and pituitary tumours. *MEN1* mutations also cause familial isolated primary hyperparathyroidism (FIHP), a milder condition causing hyperparathyroidism only. Identical mutations can cause either MEN1 or FIHP in different families, thereby implicating a role for genetic modifiers in altering phenotypic expression of tumours. We therefore investigated the effects of genetic background and potential for genetic modifiers on tumour development in adult *Men1^+/-^* mice, which develop tumours of the parathyroids, pancreatic islets, anterior pituitary, adrenal cortex and gonads, that had been backcrossed to generate C57BL/6 and 129S6/SvEv congenic strains. A total of 275 *Men1^+/-^* mice, aged 5–26 months were macroscopically studied, and this revealed that genetic background significantly influenced the development of pituitary, adrenal and ovarian tumours, which occurred in mice over 12 months of age and more frequently in C57BL/6 females, 129S6/SvEv males and 129S6/SvEv females, respectively. Moreover, pituitary and adrenal tumours developed earlier, in C57BL/6 males and 129S6/SvEv females, respectively, and pancreatic and testicular tumours developed earlier in 129S6/SvEv males. Furthermore, glucagon-positive staining pancreatic tumours occurred more frequently in 129S6/SvEv *Men1^+/-^* mice. Whole genome sequence analysis of 129S6/SvEv and C57BL/6 *Men1^+/-^* mice revealed >54,000 different variants in >300 genes. These included, *Coq7*, *Dmpk*, *Ccne2*, *Kras*, *Wnt2b*, *Il3ra* and *Tnfrsf10a*, and qRT-PCR analysis revealed that *Kras* was significantly higher in pituitaries of male 129S6/SvEv mice. Thus, our results demonstrate that *Kras* and other genes could represent possible genetic modifiers of *Men1*.

## Introduction

Multiple endocrine neoplasia type 1 (MEN1) is an autosomal dominant disorder characterised by the occurrence of parathyroid, pancreatic islet and anterior pituitary tumours. In addition, some patients may also develop adrenal cortical tumours, carcinoids, facial angiofibromas, collagenomas and lipomas (1). MEN1-associated tumours show a loss of heterozygosity of the *MEN1* gene, which is located on chromosome 11q13 and encodes the ubiquitously expressed, predominantly nuclear scaffold tumour-suppressor protein, menin (2, 3, 4). Over 1500 *MEN1* mutations have been reported, and 97% of these are associated with the simultaneous occurrence of the many tumours of the MEN1 syndrome, while the remaining 3% of mutations are associated with familial isolated hyperparathyroidism (FIHP), a disorder characterised by the sole occurrence of parathyroid tumours (5). Thirty such *MEN1* mutations have been reported in patients with FIHP, and 15 of these mutations are identical to those reported in MEN1 patients and include intragenic deletions, gross deletions, intragenic insertions, missense, nonsense and splice site mutations (4, 5); thereby indicating that the same *MEN1* mutations may cause MEN1 or FIHP in unrelated families. Overall, these findings implicate a role of modifier genes in altering the expression of *MEN1* mutations (6, 7).

Genetic modifiers have been identified to influence the phenotypic manifestation of human diseases, as illustrated by studies of patients with DiGeorge syndrome type 1 (DGS1) (8). Patients with DGS1 typically suffer from hypoparathyroidism, immunodeficiency due to thymic aplasia, congenital heart defects and deformities of the ear, nose and mouth (9). Approximately 30% of patients may also have neurodevelopmental anomalies and urogenital malformations including unilateral agenesis, renal dysplasia, hydronephrosis and uterine didelphys with duplication of the cervix (8, 10, 11). DGS1 is associated with deletions of chromosome 22q11.2, and abnormalities of T-box transcription factor 1 (TBX1) are found in >95% of DGS1 patients, although these do not explain the phenotypic variability observed in the renal and urinary tract abnormalities. However, additional studies revealed that a major driver of renal disease in DGS1 is CRK-like proto-oncogene, adaptor protein (CRKL), mutations of which sensitise the genetic background and modify the penetrance of congenital kidney and urinary tract anomalies in DGS1 patients (8).

In addition, studies of mutant mouse models for human disorders have also identified roles for genetic modifiers, in affecting the penetrance, dominance, expressivity and pleiotrophy of disease manifestations (12, 13). For example, studies of mutant mouse models have revealed that the secretory type II phospholipase A2 (*Pla2s*) gene is a major modifier of the adenomatous polyposis coli (*APC*) gene, such that its absence is associated with increased numbers of intestinal polyps in *APC* mutant mice that are on a C57BL/6J background, which are null for *Pla2s* activity, when compared to the APC mutant mice on MA/MyJ or Mus castaneus (CAST) backgrounds that highly express *Pla2s* (average number of intestinal polyps C57BL/6J:MA/MyJ:CAST = 28.5:5.7:3.0) (14). Furthermore, embryonic lethality and survival in mice associated with null mutations of several genes have been shown to be strain dependent, and studies of these mice have allowed mapping of modifier loci, for example, investigation of: transforming growth factor beta 1 null mice (*Tgfb1^-/-^*), which have vascular defects similar to those in patients with hereditary haemorrhagic telangiectasia, bred on NIH/Ola and C57BL/6J/Ola backgrounds revealed the presence of a major codominant modifier gene for embryonic lethality on mouse proximal chromosome 7 (15); cystic fibrosis transmembrane conductance regulator null mice (*Cftr^M1HSC^*/*Cftr^M1HSC^*), which usually died of intestinal obstruction similar to that observed in patients with cystic fibrosis, bred on C57BL/6J and BALB/cJ backgrounds, revealed a modifier on mouse chromosome 7 that was associated with prolonged survival, likely due to a partial rectification of the Na^+^ and Cl^-^ transport abnormalities (16); and p53 null mice (p53^-/-^) which develop multiple tumours similar to those in patients with the Li-Fraumeni syndrome (e.g. soft tissue sarcomas, osteosarcomas, breast cancer, brain tumours, leukaemia and adrenocortical carcinomas), bred on CE/J and 129/Sv backgrounds revealed the presence of a modifier for embryonic lethality on mouse chromosome 11 (17).

The influence of genetic background on the phenotypes of embryonic lethality and neural defects has also previously been reported in homozygous *Men1^-/-^* mouse embryos (18), implicating a role for genetic modifiers in MEN1 syndrome. Survival time of *Men1^-/-^* embryos was found to be significantly lower in the 129S6/SvEv strain compared with the C57BL/6 strain and neural tube defects were exclusively found in the 129S6/SvEv embryos, while widespread oedema was specific to the C57BL/6 strain (18). However, the influence of genetic background and potential role of genetic modifiers on the development of tumours in adult *Men1^+/-^* mice have not been previously studied. Identification of such modifiers of tumour expression could provide a better understanding of the function of menin and its molecular interactions in endocrine tumourigenesis. We have previously established a conventional mouse knockout model of MEN1 on a mixed 129S6/SvEv and C57BL/6 background, whereby *Men1^+/-^* mice develop tumours of the parathyroids, pancreatic islets, anterior pituitary, adrenal cortex and testes or ovaries by the age of 12 months (19). We therefore utilised this model to investigate the role of genetic background on tumour formation in adult *Men1^+/-^* mice, by carrying out backcrosses to generate *Men1^+/-^* mice on congenic C57BL/6 and 129S6/SvEv strain backgrounds.

## Materials and methods

### Generation of *Men1^+/-^* congenic mouse strains and assessments of their genotypes and phenotypes

Mice were kept in accordance with UK Home Office guidelines and project license restrictions. *Men1^+/-^* /*Men1^+/+^* mouse crosses were used as *Men1^-/-^* mice are not viable (18, 19). Genotypes of mice were determined by PCR analysis using DNA extracted from tail or ear biopsies and *Men1* gene-specific primers, as previously reported (20). Primers Men1F (5′-TAGATGTAGCTGGATGGTGATGG-3′) and Men1R (5′-ATGAAGCTGAGGAGATGATGTAG-3′) yielded a 582 base-pair WT fragment and primers Men1F and NeoR (5′-GCTGACCGCTTCCTCGTG-3′) yielded a 809 base-pair mutant fragment (Supplementary Fig. 1, see section on supplementary materials given at the end of this article). In total, 2358 mice were generated, and these comprised breeding cohorts and all genotypes from the background strains. A subset of these *Men1^+/-^* mice and *Men1^+/+^* littermates were aged to 5 to 26 months and complete necropsy was performed. At necropsy, endocrine organs, including the pituitary, pancreas, adrenal, ovaries and testes, were inspected for abnormalities, as previously described (19). Macroscopic appearances were recorded and measurements of tumours or other abnormal masses made. Tissues were dissected and fixed in 4% paraformaldehyde (PFA) for 24 h, for histological and immunohistochemical analysis, as previously described (19). Endocrine organs with and without gross abnormalities were collected separately. Parathyroid tumours were not included in this study, as owing to their small size they cannot be detected by macroscopic examination.

### Histology and immunohistochemistry

Paraffin embedded sections were dewaxed and rehydrated prior to staining. For H&E staining, haematoxylin (Modified Mayer’s Formula, Vector Laboratories, Peterborough, UK) was applied, followed by counterstaining with 1% eosin and permanent mounting (Vector Laboratories mounting media), as previously described (19). For immunohistochemical staining, antigen retrieval was performed using citrate buffer (0.1 M, pH 6.0) or High pH Antigen Retrieval Solution (Vector Laboratories), with heating (autoclave; 121°C for 10 min) or a combination of heating and pressure (antigen decloaking chamber). Tissue was blocked using 0.3% hydrogen peroxide/methanol and 10% serum from the secondary antibody host. Primary antibodies included: anti-menin (AbCam ab2605); anti-prolactin (National Hormone and Peptide Programme, Torrance, CA, USA); anti-growth hormone (AbCam ab8490); anti-chromogranin A (AbCam ab301704); anti-insulin (AbCam ab7842); anti-glucagon (AbCam ab10561971) and anti-Kras (AbCam ab84573). All HRP-conjugated secondary antibodies (Jackson Laboratories) were applied for 1 h, followed by 3,3′-diaminobenzidine (DAB) substrate (Vector Laboratories) for <5 min, and sections were counterstained with haematoxylin, as previously described (21). Sections were imaged and captured using an Eclipse E400 microscope (Nikon) and DXM1200C digital camera and NIS-Elements BR 2.30 software (both Nikon) (19, 22).

### Whole genome sequence analysis

DNA was extracted from ear biopsies of C57BL/6J and 129S6/SvEv mice using the Blood Core II kit (Qiagen) and used to generate a library for whole genome sequencing (WGS) that ustilised the Illumina HiSeq platform at the Oxford Genomics Centre (Wellcome Trust Centre for Human Genetics, University of Oxford), and single nucleotide variants called, as previously described (23). The generated gene lists were analysed using the Protein Analysis Through Evolutionary Relationship (PANTHER) system (http://pantherdb.org) (24).

### Quantitative reverse transcription PCR (qRT-PCR)

Pituitary, ovary and testes tissues were harvested and placed immediately into RNAlater solution (Life Technologies). Pancreatic islets were picked from pancreatic tissue digested following direct intra pancreatic injection of 2 mL of 0.2 mg/mL Liberase (Roche) and placed immediately into RNAlater solution (25). Total RNA was extracted from the tissues using the RNeasy kit (Qiagen), and up to 1µg of total RNA was used to generate cDNA using the Quantitect RT kit (Qiagen), as described (26). Quantitect primers (Qiagen) were used for qRT-PCR reactions, which utilised the Quantitect SYBR green kit (Qiagen), on a RotorGene 5 (Qiagen), as described (26). Each test sample was normalized to the geometric mean of reference genes GAPDH, calnexin and α-tubulin. The relative expression of target cDNA in all qRT-PCR studies was determined using the Pfaffl method (27).

### Western blot analysis

Mouse pituitaries were lysed in NP40 lysis buffer and prepared in 4× Laemmli loading dye, as previously described (26). Samples were resolved using 10% SDS-PAGE gel electrophoresis, transferred to polyvinylidene difluoride membrane, probed with primary antibodies (Calnexin-AB2301 (Millipore), Kras-ab84573 (AbCam)) and an anti-rabbit HRP-conjugated secondary antibody (Santa Cruz Biotechnology) and then visualised using Pierce ECL Western Blotting substrate (Thermo Fisher Scientific), as previously described (26). Calnexin protein expression was used as a loading control. Densitometry analysis was performed by calculating the number of pixels per band using ImageJ software (NIH). Data were represented as the number of pixels of the protein band, relative to the number of pixels of the corresponding calnexin band.

### Statistical analysis

GraphPad Prism was used for the statistical analyses and the generation of graphs. The total proportion of *Men1^+/-^* mice with tumours, including tumour subtypes, was compared between two different genetic backgrounds by performing a Fisher’s exact test, with the threshold for statistical significance set at *P* < 0.05. A Bonferroni correction was applied to account for multiple tests. To evaluate the trend of tumour development, a Chi squared test for trends was applied. For all other analyses one-way ANOVA or *t*-tests were used, as previously described (26).

## Results

### Development of tumours in congenic *Men1^+/-^* mice

**Figure 1:**
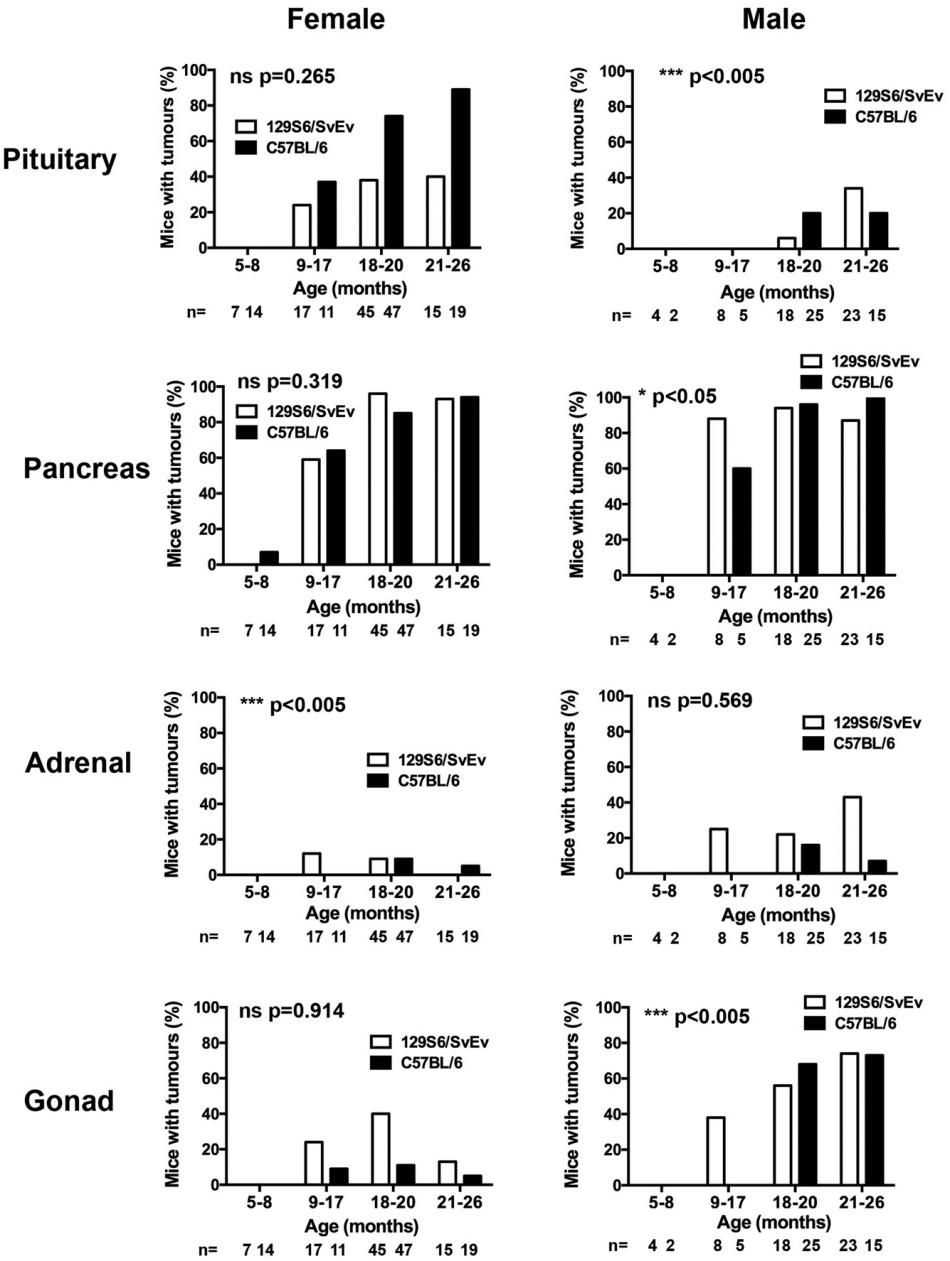
Age of tumour occurrence in endocrine organs of male and female congenic *Men1^+/-^* mice. The pituitary, pancreas, adrenals and gonads, which have previously been reported (19) to have significantly altered tumour development in 5- to 26-month-old 129S6/SvEv and C57BL/6 *Men1^+/-^* mice, were macroscopically examined for tumour development in four different age groups (5–8 months, 9–17 months, 18–20 months and 21–26 months) of *Men1^+/-^* mice, with the number (*n*) of mice per group. Differences in the of age of tumour development in 129S6/SvEv compared to C57BL/6 *Men1^+/-^* mice were assessed by Chi-squared test for trend.

A total of 2358 mice (1166 *Men1^+/+^* and 1192 *Men1^+/-^* mice) on 129S6/SvEv (*n* = 1166) and C57BL/6 (*n* = 1192) genetic background strains and backcross generations 10–18 were produced. There were no significant differences in the live births or survival to age 18–21 months between the *Men1^+/-^* or *Men1^+/+^* mice or between the background strains (live births:* Men1^+/+^* (*n* = 600 129S6/SvEv; *n* = 566 C57BL/6) vs *Men1^+/-^* (*n* = 566 129S6/SvEv; *n* = 626 C57BL/6); and survival: *Men1^+/+^* (*n* = 146 129S6/SvEv; *n* = 95 C57BL/6) vs *Men1^+/-^* (*n* = 135 129S6/SvEv; *n* = 149 C57BL/6) (Supplementary Tables 1 and 2)). From these, a total of 275 *Men1^+/-^* mice from 129S6/SvEv (*n* = 137) and C57BL/6 (*n* = 138) genetic background strains, aged 5–26 months, were macroscopically assessed for the prevalence of endocrine tumours in the pancreas, pituitary, adrenals and gonads. This age range was chosen as our previous study indicated that mice develop a significant number of tumours after 12 months of age, although tumours can be identified as early as 3 months of age (19). The number of tumours that developed and the organs affected increased with the age of the mice, consistent with the manifestations of the MEN1 syndrome in patients. However, the frequency of tumour development and age of onset depended on the background strain and sex of the mice. Thus, overall, more pituitary tumours developed in female C57BL/6 *Men1^+/-^* mice than in female 129S6/SvEv *Men1^+/-^* mice, while more adrenal tumours developed in male 129S6/SvEv *Men1^+/-^* than in male C57BL/6 *Men1^+/-^* mice and more ovarian tumours developed in 129S6/SvEv *Men1^+/-^* mice than in C57BL/6 *Men1^+/-^* mice (Table 1). When examined by age, significant differences were identified in the age of onset of these tumours, with: pituitary tumours occurring significantly earlier in male C56BL/6 *Men1^+/-^* mice (*P* < 0.005), when compared to 129S6/SvEv *Men1^+/-^* mice; pancreatic tumours occurring significantly earlier in male 129S6/SvEv than in C57BL/6 *Men1^+/-^* mice (*P* < 0.05); adrenal tumours occurring significantly earlier in female 129S6/SvEv *Men1^+/-^* mice than in C57BL/6 *Men1^+/-^* mice (*P* < 0.005) and testicular tumours occurring significantly earlier in male 129S6/SvEv *Men1^+/-^* than in C57BL/6 *Men1^+/-^* mice (*P* < 0.005) (Fig. 1). The occurrence of these tumours is described in more detail subsequently.

**Table 1:** Occurrence of endocrine tumours observed in 12- to 26-month-old *Men1^+/-^* mice in 129S6/SvEv and C57BL/6 strains.

Tumours	Strain		*P*
	129S6/SvEv	C57BL/6	
Pituitary			
F	27/71 (38.0%)	56/74 (75.7%)	<0.0001
M	9/45 (20.0%)	8/43 (18.6%)	NS
Pancreas			
F	66/71 (93.0%)	62/74 (83.8%)	NS
M	41/45 (91.1%)	41/43 (95.3%)	NS
Adrenal			
F	7/71 (9.9%)	6/74 (8.1%)	NS
M	14/45 (31.1%)	5/43 (11.6%)	0.0376
Ovaries			
F	23/71 (32.4%)	8/74 (10.8%)	0.0021
Testes			
M	29/45 (64.4%)	28/43 (65.1%)	NS

Data are shown as the number of mice with tumours/total number of mice examined, with the percentage in parenthesis. Results are based on macroscopic findings at necropsy.

F = female; M = male; NS = not significant.

### Pituitary tumours in 129S6/SvEv and C57BL/6 *Men1^+/-^* mice

**Figure 2:**
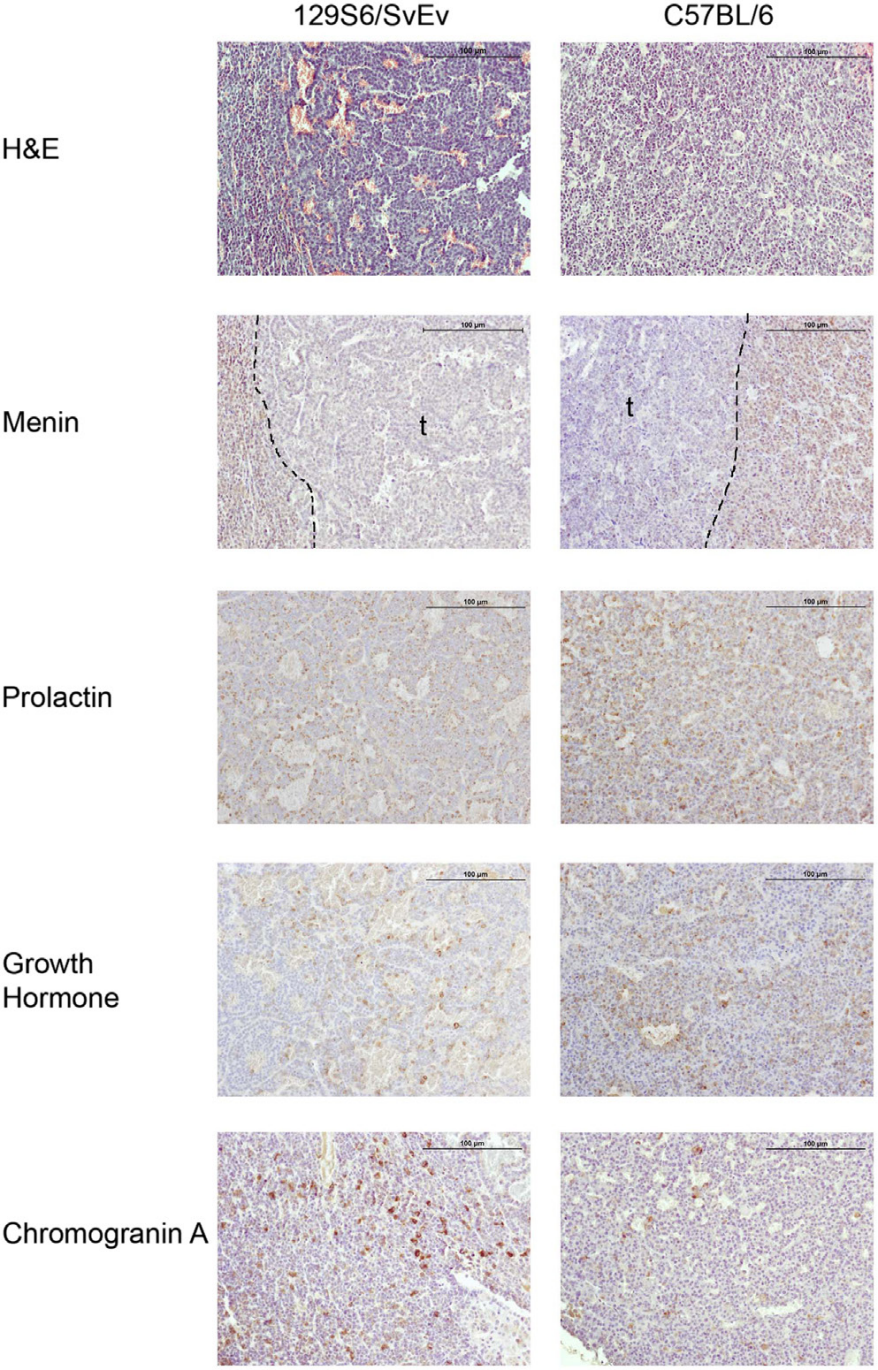
Histological and immunohistochemical analysis of anterior pituitary tumours in congenic *Men1^+/-^* mice. Serial sections of anterior pituitary adenomas from 18-month-old *Men1^+/-^* 129S6/SvEv and C57BL/6 mice were stained with haematoxylin and eosin (H&E) and for menin, prolactin, growth hormone and chromogranin A, which is a neuroendocrine secretory protein (44). Menin expression was lost within the pituitary adenomas (t), whereas prolactin, growth hormone and chromogranin A, identified by brown DAB staining, were all expressed within cells of the anterior pituitary adenomas in both mouse strains. These findings indicate that the tumours are adenomas originating from the anterior pituitary and that they are associated with loss of menin expression. The scale bar represents 100 μM.

Pituitary tumours were observed in the *Men1^+/-^* mice at necropsy, from the age of 13 months in the 129S6/SvEv strain and from 15 months of age in the C57BL/6 strain (Fig. 1). Histological and immunohistochemical analysis confirmed loss of menin expression in pituitary tumours (*n* = 4) from C57BL/6 *Men1^+/-^* and 129S6/SvEv *Men1^+/-^* mice and expression of prolactin, growth hormone and chromogranin A (Fig. 2). The anterior pituitary tumours were significantly more frequent in the female *Men1^+/-^* mice than in male *Men1^+/-^* mice (129S6/SvEv females vs males = 38% vs 20.0%, *P* < 0.005, and C57BL/6 females vs males = 75.7% vs 18.6%, *P* < 0.0001) (Table 1). In addition, significantly more C57BL/6 female *Men1^+/-^* mice had pituitary tumours than 129S6/SvEv female *Men1^+/-^* mice (75.7% vs 38.0%, *P* < 0.0001) (Table 1), whereas there was no significant difference in the number of anterior pituitary tumours in male *Men1^+/-^* mice between the two strains (Table 1).

### Pancreatic neuroendocrine tumours in 129S6/SvEv and C57BL/6 *Men1*^+/-^ mice

**Figure 3:**
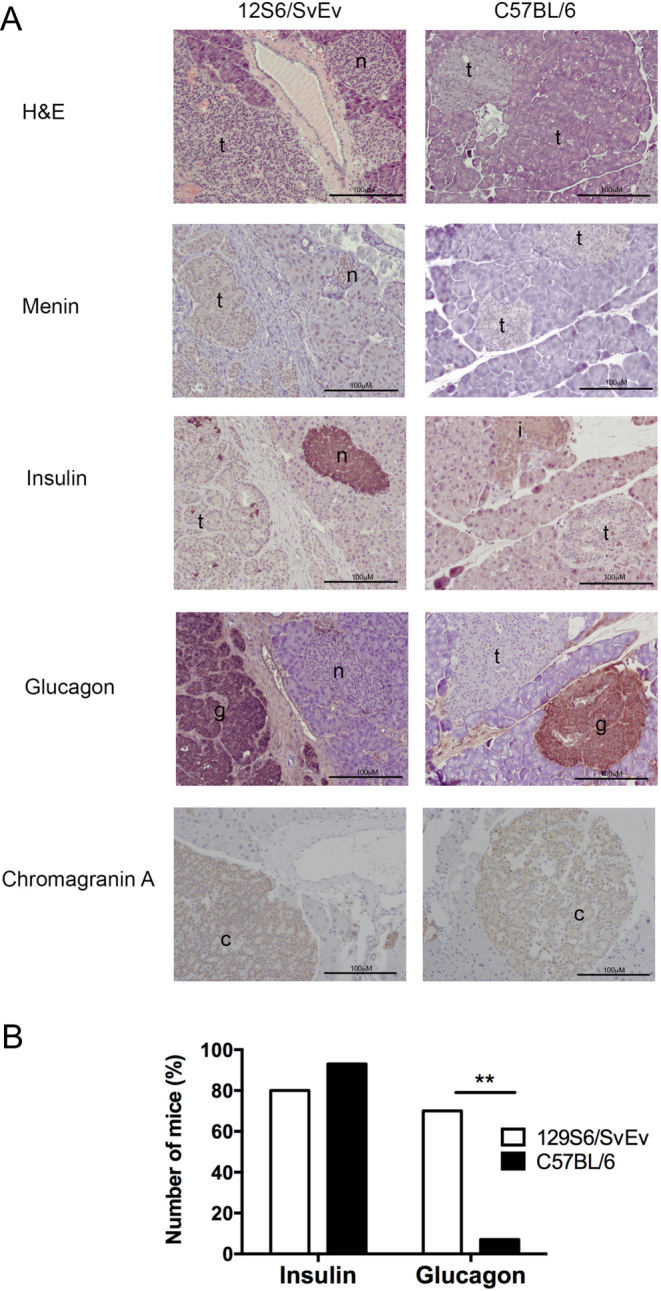
Histological and immunohistochemical analysis of PNETs in congenic *Men1^+/-^* mice. (A) Serial sections of pancreatic islet tumours from 25 ≥18-month-old *Men1^+/-^* 129S6/SvEv and C57BL/6 mice were stained with haematoxylin and eosin (H&E) and for menin, insulin, glucagon, and chromogranin A. Menin expression, indicated by brown DAB staining, was observed in normal islets (*n*) and normal surrounding exocrine tissue, whereas pancreatic islet adenomas showed loss of menin expression (t). Tumours with loss of menin expression (t), predominantly expressed either insulin (i) or glucagon (g). The tumours also immunostained for chromogranin A (c), confirming their neuroendocrine origin. The scale bar represents 100 μM. (B) There was no significant difference in the development of insulin-expressing PNETs between 129S6/SvEv *Men1^+/-^* mice and C57BL/6 *Men1^+/-^* mice, but a significantly greater number of 129S6/SvEv *Men1^+/-^* mice developed glucagon-expressing PNETs, when compared to C57BL/6 *Men1^+/-^* mice (**P < 0.005 using a Fishers exact test).

Pancreatic neuroendocrine tumours (PNETs) were the most frequent tumour type observed at necropsy and were detected in >85% of 129S6/SvEv and C57BL/6 *Men1^+/-^*mice (Table 1). The occurrence of PNETs was not significantly different between the two strains or between sexes (Table 1). A total of 25 PNETs (10 from 129S6/SvEv (*n* = 4 male and *n* = 6 female) and 15 from C57BL/6 (*n* = 2 male and *n* = 14 female) *Men1^+/-^*mice were analysed by histology and immunohistochemistry, which confirmed loss of menin expression, but presence of chromogranin A expression (Fig. 3A). This analysis also revealed the occurrence of multiple PNETs (range = 1–9 PNETs per mouse) that varied in size in the 129S6/SvEv and C57BL/6 *Men1^+/-^*mice. Some tumours expressed insulin, while others expressed glucagon (Fig. 3A), and one C57BL6 and six 129SvEv *Men1^+/-^*mice had simultaneous occurrence of insulin and glucagon-expressing tumours, although tumours co-expressing both hormones were not found. There was no significant difference in the occurrence of insulin-expressing PNETs between the two mouse strains (129S6/SvEv *Men1^+/-^* vs C57BL/6 *Men1^+/-^* mice = 80% vs 93%) (Fig. 3B). However, glucagon-expressing tumours developed significantly more frequently in the 129S6/SvEv *Men1^+/-^* mice than in C57BL/6 *Men1^+/-^* mice (7/10 mice vs 1/15 mice, respectively; *P* < 0.002, Fig. 3B), such that glucagon-expressing tumours of the 129S6/SvEv *Men1^+/-^* mice accounted for 37% (13/35) of the total PNETs, whereas glucagon-expressing tumours of C57BL/6 *Men1^+/-^* mice accounted for only 2% (1/43) of all PNETs (*P* < 0.0001).

### Adrenal tumours in 129S6/SvEv and C57BL/6 *Men1*^+/-^ mice

**Figure 4:**
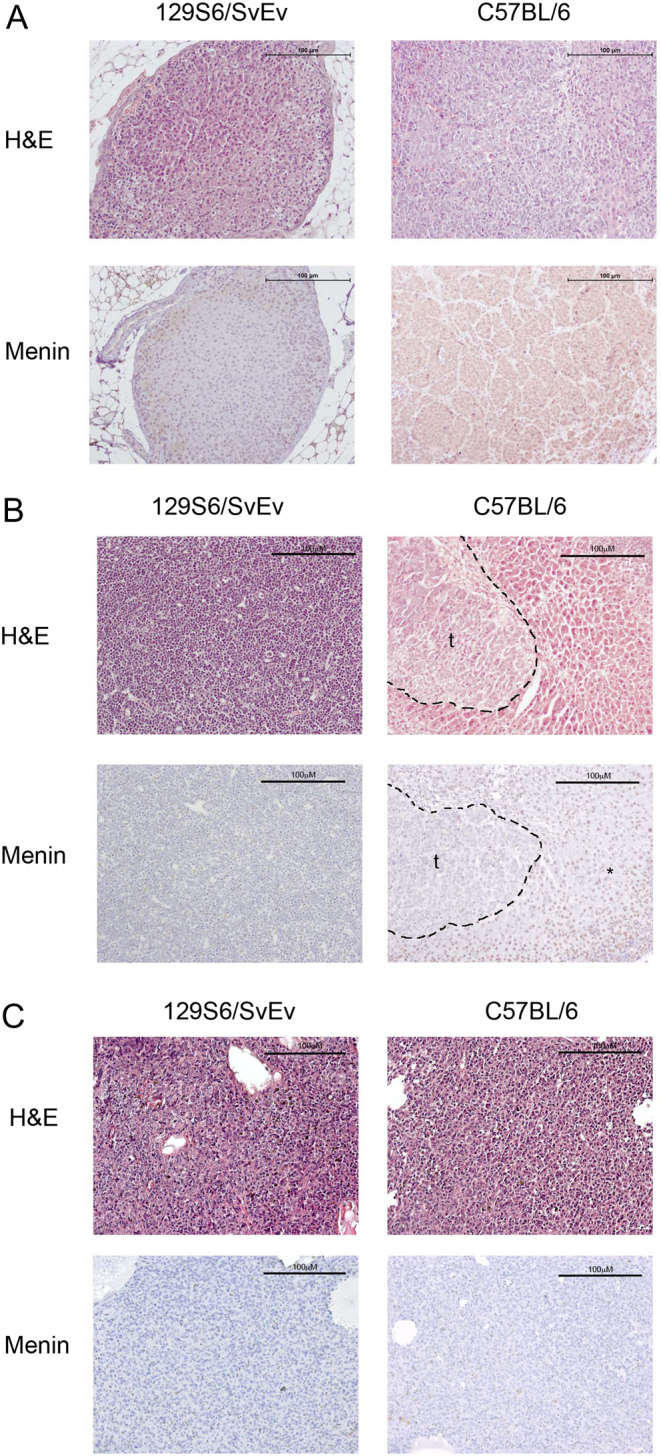
Histological and immunohistochemical analysis of adrenal, ovarian and testicular tumours from congenic *Men1^+/-^* mice. (A) Adrenal cortical tumours from 23-month-old male 129S6/SvEv *Men1^+/-^* and C57BL/6 *Men1^+/-^* mice were stained with haematoxylin and eosin (H&E) and for menin. In both strains, menin expression was lost in the tumours. The scale bar represents 100 μM. (B) Ovarian tumours from 18-month-old female *Men1^+/-^* 129S6/SvEv and C57BL/6 mice were stained with H&E and menin. Menin, identified by brown nuclear DAB staining (*), was lost in the tumours (t) and retained in the surrounding normal tissue. The scale bar represents 100 μM. (C) Testicular tumours from 18-month-old male *Men1^+/-^* 129S6/SvEv and C57BL/6 mice were stained with H&E and menin. In both strains, menin expression was lost in the tumours. The scale bar represents 100 μM.

Adrenal tumours were detected from 12 to 15 months of age in male and female 129S6/SvEv *Men1^+/-^* mice, respectively, but from 19 and 18 months of age in male and female C57BL/6 *Men1^+/-^* mice, respectively, thereby indicating a significant earlier age for development of adrenal tumours in the 129S6/SvEv *Men1^+/-^* mice than the C57BL/6 *Men1^+/-^* mice (*P* < 0.0005, Fig. 1). Furthermore, the occurrence of adrenal tumours in male 129S6/SvEv *Men1^+/-^* mice was significantly higher than that in the male C57BL/6 *Men1^+/-^* mice (31.1% vs 11.6%, respectively, *P* < 0.05; Table 1), while the overall occurrence of adrenal tumours in female *Men1^+/-^* mice was similar at all ages between both strains (Table 1). Immunohistochemical analysis of adrenal tumours (*n* = 4) indicated these tumours to arise from the adrenal cortex and confirmed loss of menin expression (Fig. 4A).

### Gonadal tumours in 129S6/SvEv and C57BL/6 *Men1^+/-^* mice

Ovarian tumours were detected from 12 months of age in 129S6/SvEv *Men1^+/-^* mice and from 17 months of age in C57BL/6 *Men1^+/-^* mice, which was not statistically significantly different (Fig. 1). However, the occurrence of ovarian tumours was higher in 129S6/SvEv *Men1^+/-^* mice compared to that in the C57BL/6 *Men1^+/-^* mice (32.4% vs 10.8%, respectively, *P* < 0.005; Table 1). Immunohistochemical analysis confirmed the loss of menin in these tumours (Fig. 4B). The occurrence of testicular tumours was not statistically significant different between the two strains (Table 1), but they were detected earlier in male 129S6/SvEv *Men1^+/-^* mice than C57BL/6 *Men1^+/-^* mice (12 vs 18 months of age (*P* < 0.005, Fig. 1). Immunohistochemical analysis confirmed the loss of menin in these tumours (Fig. 4C).

### Genetic variants present in C57BL/6 *Men1^+/-^* mice compared to 129S6/SvEv *Men1^+/-^* mice

**Figure 5:**
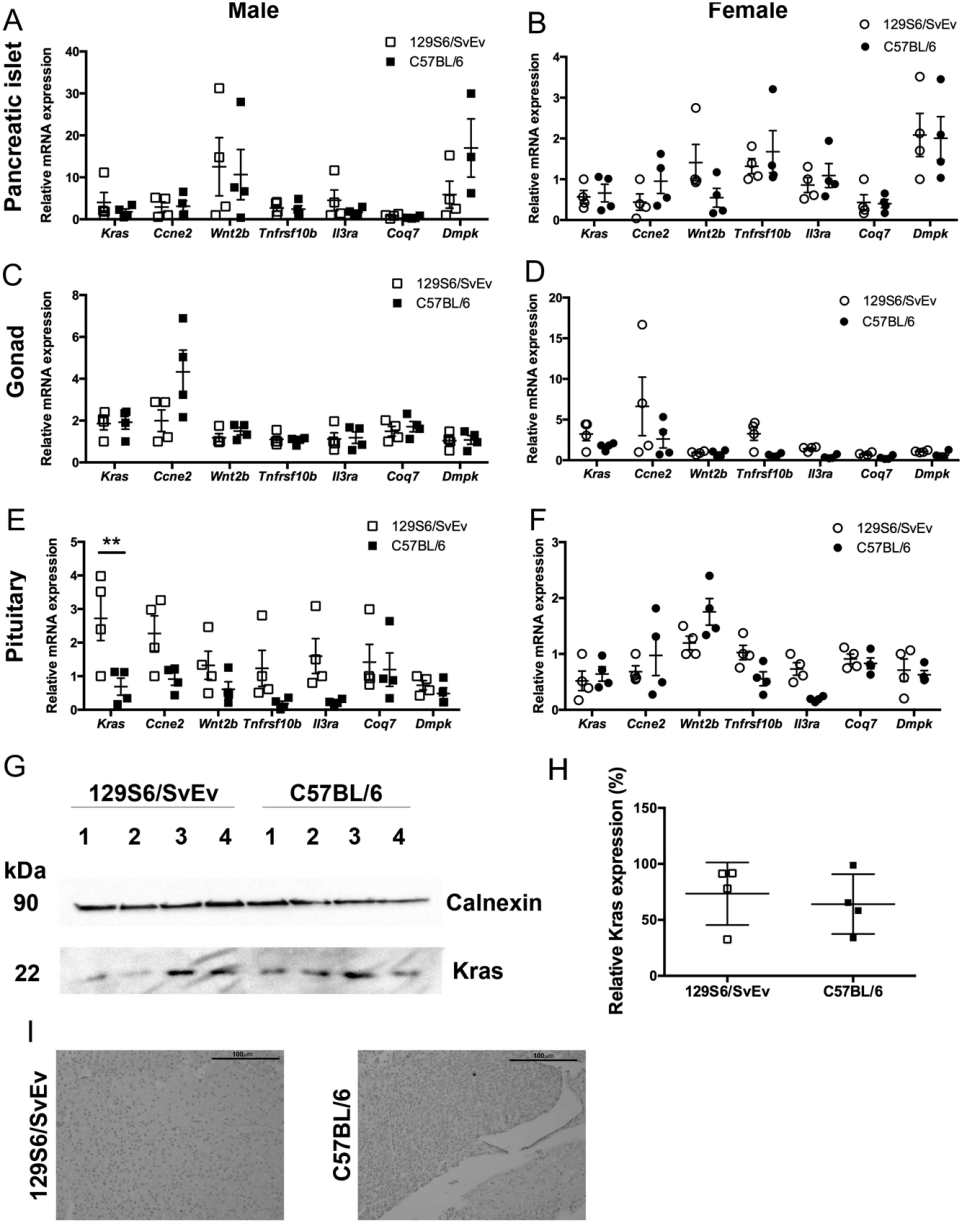
Expression of tumour-associated pathway genes with sequence variants observed in 129S6/SvEv and C57BL/6 mice. (A, B, C, D, E, and F) qRT-PCR was used to evaluate the expression of seven genes (*Kras*, *Ccne2*, *Wnt2b*, *Tnfrsf10b*, *Il3ra*, *Coq7* and *Dmpk*) in pancreatic islet (A and B), gonadal (C and D) and pituitary (E and F) tissue harvested from male and female WT (*Men1^+/+^*) 129S6/SvEv and C57BL/6 mice. Data are represented as mean and s.e.m.; relative to one 126S6/SvEv mouse; *n* = 4 mice per group; **P < 0.005. (G) Western blot analysis of KRAS expression in male pituitary tissue harvested from *Men1^+/+^* 129S6/SvEv and C57BL/6 mice; calnexin was used as a housekeeper. (H) KRAS expression from the Western blots was quantified using densitometry analysis. Data are represented as mean and s.e.m.; *n* = 4 mice per group. (I) Immunohistochemical analysis of KRAS expression in sections of male *Men1^+/+^* 129S6/SvEv and C57BL/6 mice showing very low expression in PNET tissues. All images were taken using a ×20 objective, with the scale bar representing 100 μM.

To identify potential genetic modifiers contributing to the observed phenotypic differences in the C57BL/6 vs 129S6/SvEv *Men1^+/-^* mice, WGS was performed. A total of 54,845 genetic variants in 304 different genes were identified between C57BL/6 and 129S6/SvEv *Men1^+/-^* mice (Table 2). The variants were located in exons, introns (including splice regions), 3′ UTRs and 5′ UTRs, as well as upstream and downstream of gene loci (Table 2). Variants in the exonic regions included two nonsense variants in the Coenzyme Q7 (*Coq7*, Trp226Stop (W226*)) and dystrophia myotonica protein kinase (*Dmpk*, Tyr558Stop (Y558*)) genes, which were present in 129S6SvEv *Men1^+/-^* mice but not C57BL/6 *Men1^+/-^* mice. However, assessment of evolutionary conservation across mouse and human genomes revealed that the location of the variant in *Coq7* only occurs in one transcript that is not present in humans and that the C terminal of the encoded protein from the mouse *Dmpk* transcript (547–582) only shares 14% identity with the human protein. This lack of conservation indicates that these variants are unlikely to be genetic modifiers of the *MEN1* gene. In addition to the nonsense variants, 285 missense variants, 217 5′ UTR variants, 123 splice region variants and 1287 3′ UTR variants were identified within 216 different genes. PANTHER analysis was used to identify the functional classification of these genes, and this indicated that variants were present in many genes within tumour-associated cellular pathways including: cell cycle regulation (cyclin E2 (*Ccne2*)); Ras signalling (Kirsten rat sarcoma viral oncogene homologue (*Kras*)); wnt signalling (wingless-type MMTV integration site family member 2B (*Wnt2b*)); interleukin signalling (Interleukin-3 receptor subunit alpha (*Il3ra*)) and apoptosis signalling (TNF receptor superfamily member 10a (*Tnfrsf10a*)) (Supplementary Table 3). These, together with *Coq7* and *Dmpk*, were therefore selected for further study. The expression of these genes was evaluated by qRT-PCR in pituitary, pancreatic islets and gonadal tissues from *Men1^+/+^* C57BL/6 and 129S6/SvEv mice. This did not reveal significant differences in the expression of any of these genes in the pancreatic islets (Fig. 5A and B) or gonadal tissue (Fig. 5C and D) in either male or female 129S6/SvEv mice, when compared to C57BL/6 mice. In the pituitary, no significant difference was observed in *Ccne*, *Wnt2b*, *Il3ra*, *Tnfrsf10a*, *Coq7* or *Dmpk* expression in either male or female 129S6/SvEv mice, compared to C57BL/6 mice (Fig. 5A and B). However, significantly higher expression of *Kras* was detected in the pituitaries of male 129S6/SvEv *Men1^+/+^* mice, when compared to male C57BL/6 *Men1^+/+^* mice (4-fold, *P* < 0.005, Fig. 5E); such significant differences were not detected in *Kras* expression in female *Men1^+/+^* mice (Fig. 5F). These findings suggest that *Kras* may be a potential modifier of *Men1*, and it is interesting to note that KRAS has been reported to suppress growth in pancreatic endocrine cells and that this is influenced by expression of menin (28). We therefore further investigated KRAS protein expression in the pituitaries of male *Men1^+/+^* 129S6/SvEv and C57BL/6 mice by Western blot and immunohistochemistry analyses. Overall expression of the KRAS protein was very low and significant differences in KRAS protein expression were not detected (Fig. 5G, H and I).

**Table 2:** Genetic variants identified in C57BL/6 *Men1^+/-^* mice compared to 129S6/SvEv *Men1^+/-^* mice, by whole genome sequencing. In total 54,845 variants were identified.

Variant type	Number of variants
5′ UTR^a^	217
Intronic/intergenic	46,206
Splice region	123
Exonic	
Non-coding	13
Synonymous	574
Missense	285
Nonsense	2
3′ UTR^a^	1287
Upstream gene	3221
Downstream gene	2917
Total	54,845

^a^UTR, untranslated region.

## Discussion

Our results reveal that genetic background significantly influenced the development of pituitary (Fig. 1 and Table 1), glucagon-expressing pancreatic (Fig. 3), adrenal and gonadal tumours (Fig. 1 and Table 1) in *Men1^+/-^* mice, which represent a model for the MEN1 syndrome, thereby supporting the role for genetic modifiers in altering the phenotype of this autosomal dominant disorder. These genetic modifiers may form part of the tumourigenic pathways, as suggested by our WGS analysis, and further analysis of these may provide important clues about the biological roles of these genes in cell cycle regulation and apoptosis in endocrine cells. In addition, identification of these genetic modifiers and their roles may help to provide explanations for the heterogeneity and age-related penetrance of MEN1, as well as identifying tumour-associated pathways that may be targeted by anti-proliferative compounds.

Our studies of genetic background on endocrine tumour formation in adult *Men1^+/-^* mice has revealed that the tumours observed and their age of onset, in the 129S6/SvEv and C57BL/6 congenic *Men1^+/-^* mouse strains, were comparable to that in the same model on a mixed 129S6/SvEv and C57BL/6 background, with less than five consecutive backcrosses since the generation of the original chimera (19) to that described in other reported *Men1* conventional mouse knockout models on a pure 129S6/SvEv background (29) or mixed backgrounds (Supplementary Table 4) (19, 30, 31). In each model, pancreatic NETs developed earliest and most frequently, with all models also developing the range of tumours seen in our congenic strains, with the exception of one model, which was on a mixed NIH Black Swiss and 129/SvEvTacFBR background and did not report the development of gonadal tumours (Supplementary Table 4) (31). Unlike previous reports, our study compares mouse models maintained on two different background strains and is therefore able to assess the effects of genetic background on the phenotypic manifestations of *Men1* knockout.

The frequency of pituitary tumour development in female 129S6/SvEv *Men1^+/-^* mice over 12 months of age in our study was consistent with that of another 129S6/SvEv model (29) (38% vs 32%, respectively), while the significantly increased frequency of pituitary tumours in female C57BL/6 *Men1^+/-^* mice (75.7%) is consistent with a previous C57BL/6 mouse study (77.8%) (30). Our results were also consistent with those reporting an increased occurrence of pituitary tumours in female mice, compared to males (19, 29). Thus, genetic background is an important determinant of pituitary tumour development in *Men1^+/-^* mice. We also show that tumour development is not just accelerated in the C57BL/6 model, as we observed a significant increase in the occurrence of adrenal and ovarian tumours in the 129S6/SvEv, when compared to the C57BL/6 *Men1^+/-^* mice. Differences in tumour development between strains were also not limited to one sex, since male 129S6/SvEv *Men1^+/-^* mice developed significantly more adrenal tumours than C57BL/6 *Men1^+/-^* mice, while significantly more female 129S6/SvEv *Men1^+/-^* mice developed ovarian tumours compared to female C57BL/6 *Men1^+/-^* mice. The influence of genetic background on tumour phenotype may therefore explain the differences observed in four previously reported *Men1^+/-^* conventional mouse models; for example, gastric neuroendocrine tumours were reported only in a mixed NIH Black Swiss and 129/SvEvTacFBR model (Supplementary Table 4) (19, 29, 30, 31).

We observed no significant differences in the proportion of mice developing pancreatic islet tumours; however, 129S6/SvEv mice developed significantly more glucagon-expressing tumours, which did not express insulin, than the C57BL/6 strain. In normal mouse pancreatic islets, α-cells are located on the periphery of the islet secrete glucagon, whereas β-cells are located at the centre of the islet secrete insulin, and hence, it would be expected that glucagonomas would arise from α-cells and insulinomas from β-cells. However, studies of an α-cell specific *Men1* knockout mouse model produced on a mixed genetic background that included the C57BL/6 mouse strain have reported that mice developed both glucagonomas and insulinomas (32, 33). In MEN1 patients, it has also been shown that early onset pancreatic microadenomas with loss of heterozygosity (LOH) of *MEN1* express glucagon, whereas more advanced hormone-secreting tumours in the same patients were predominantly insulinomas (34, 35). Thus, it seems possible that the *Men1^+/-^* C57BL/6 mice, in our study, had more advanced PNETs, when compared to 129S6/SvEv *Men1^+/-^* mice, which would have less advanced lesions. Detailed investigations of proliferation index using Ki67 staining or longitudinal proliferation studies using bromodeoxyuridine (BrdU) may help to clarify this. Another possibility is that the genetic background and any modifier genes may instead influence the cell types giving rise to the PNETs, and this may explain the absence of significant differences in the number of pancreatic tumours that occur at any age in the two mouse strains (Fig. 1 and Table 1).

The lack of genotype-phenotype correlation in MEN1 patients (1) may involve roles for modifier genes. Thus, loss of menin expression is a pre-requisite for tumourigenesis; however, genes involved in the same proliferative and apoptotic tumourigenic pathways may act as modifiers. Identifying such *MEN1* modifiers is of particular importance, as it may provide a tool for predicting tumour manifestations in MEN1 patients, as well as providing novel targets for both mono- and combination-drug therapies. Our WGS analysis identified >54,000 variants, within >300 genes between 129S6/SvEv *Men1^+/-^* mice and C57BL/6 mice *Men1^+/-^* mice, which could represent *Men1* genetic modifiers. *In silico* functional analysis revealed variants in genes associated with tumourigenic pathways, including *Kras*, *Wnt2b*, *Il3ra* and *Tnfrsf10a*, which were associated with Kras, wnt, interleukin and apoptosis signalling, respectively. These genes have potential to be *MEN1* modifiers, as the *Men1* protein product, menin, has been shown to: repress MAPK-driven proliferation downstream of KRAS (28), control wnt signalling through interaction with β-catenin (36, 37, 38), regulate expression of interleukins (39) and promote TNF-α induced apoptosis through up-regulation of caspase 8 (40). In addition, an association between a cyclin dependent kinase inhibitor 1B (*Cdkn1b*) variant (c.326T>G) and tumour multiplicity in MEN1 patients has been reported (41), although we did not observe any variants in *Cdkn1b* (encoding p27^kip1^) in our WGS data, we did observe variants in the cell cycle regulator, *Ccne2*, that is regulated by p27^kip1^. *Ccne2* encodes cyclin E2 which, when in complex with cyclin dependent kinase 2 (Cdk2), is inhibited by p27^kip1^, and it has been demonstrated that cyclin E-Cdk2 substrates may vary in different cell types (42). In contrast, however, it has been reported that MEN1 tumourigenesis in the pituitary and pancreatic islet requires Cdk4 but not Cdk2 (43).

Investigation of candidate modifier genes expression in WT C57BL/6 and 129S6/SvEV mice showed significant differences only in *Kras* expression in male pituitary tissue. It has been reported that KRAS can suppress pancreatic endocrine cell growth and that inhibition of proliferation by KRAS is dependent on the RAS effector protein RASSF1A and inhibition of the downstream mitogen-activated protein kinase (MAPK) signalling pathway by menin (28). Therefore, variants in KRAS could influence menin-associated proliferative pathways. Our studies revealed that *Kras* transcription was significantly decreased in C57BL/6 *Men1^+/+^* male pituitaries, when compared to pituitaries of *Men1^+/+^* male 129SvEv mice (Fig. 5E), although no significant changes could be detected in KRAS protein levels. This discrepancy may be due to variability in KRAS protein translation and protein stability. Furthermore, as changes in KRAS transcripts were only detected in the pituitary but not in the pancreatic islets or gonads (Fig. 5A, B, C, D, E and F), it is possible that different genes may modify tumour development, in a tissue-specific manner. Thus, our studies have identified possible roles for *Kras*, *Wnt2b*, *Il3ra* and *Tnfrsf10a* as potential *MEN1* genetic modifiers, and further, more detailed *in vitro* and *in vivo* studies will help to clarify their biological roles in *MEN1* tumourigenesis.

In summary, our results demonstrate that genetic background alters the phenotypic expression of PNETs and pituitary, adrenal and ovarian tumours due to loss of *Men1*, thereby providing a model that will help to improve our understanding of the clinical manifestations of *MEN1* mutations in different patients.

## Supplementary Material

Supplementary Figure 1

Supplementary Table 1. Total number of Men1+/+ and Men1+/- mouse births on C57BL/6 and 129S6/SvEv backgrounds, throughout the duration of the study, including mice generated for breeding purposes only. Data is represented as the total number of mice with statistical significance determined using a Chi-square test.

Supplementary Table 2. Survival of Men1+/+ and Men1+/- mice on C57BL/6 and 129S6/SvEv backgrounds between 18-27 months of age. Data is represented as a percentage, with the number of mice in brackets. Statistical significance was determined using a Fisher’s exact test (two tailed).

Supplementary Table 3. Variants identified in genes associated with tumourigenic pathways.

Supplementary Table 4. Multiple endocrine neoplasia type I (MEN1) conventional mouse knockout models and their background strains.

## Declaration of interest

The authors declare that there is no conflict of interest that could be perceived as prejudicing the impartiality of the research reported.

## Funding

This work was supported by the UK Medical Research Council (MRC) programme grants G9825289 and G1000462 (K E L, A A C R, M S, K G K, S E P, P T C, P N and R V T) and a Wellcome Trust Clinical Research Training Fellowship grant 087332/Z/08/Z to M J. R V T is a Wellcome Trust Investigator.
