# Supplementary material for: Genetic background influences tumour development in heterozygous Men1 knockout mice
Source: Endocr Connect. 2020 Apr 28;9(5):426–37. doi: 10.1530/EC-20-0103 (PMC7274560; doi:10.1530/EC-20-0103)
Supplement: Supplementary Figure 1 [file supplementary_figure_1.pdf]

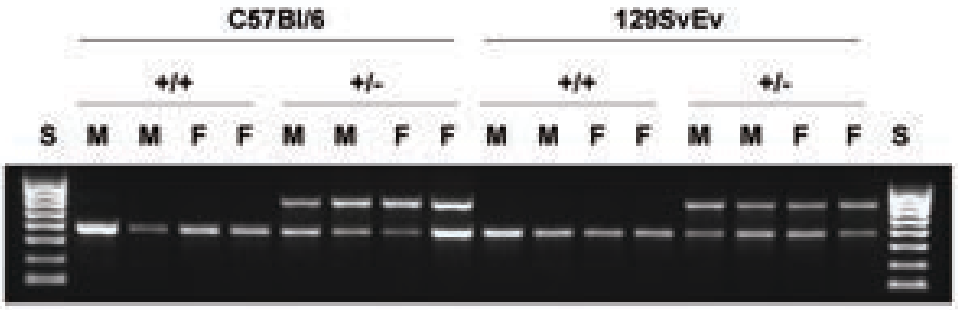

Supplementary Figure 1. *Men1* mouse genotyping. Representative genotypes of wild type ($^{+/+}$) and Menin knockout ($^{+/-}$) mice on C57Bl/6 and 129S6/SvEv genetic backgrounds. Genotypes were determined by PCR, using DNA extracted from ear biopsies. The wild type *Men1* allele is 582bp in size, and mutant *Men1* allele (resulting in loss of protein expression) is 809bp in size. S – standard; M – male; F – female.

Figure X
