# Supplementary material for: Genetic background influences tumour development in heterozygous Men1 knockout mice
Source: Endocr Connect. 2020 Apr 28;9(5):426–37. doi: 10.1530/EC-20-0103 (PMC7274560; doi:10.1530/EC-20-0103)
Supplement: Supplementary Table 1. Total number of Men1+/+ and Men1+/- mouse births on C57BL/6 and 129S6/SvEv backgrounds, throughout the duration of the study, including mice generated for breeding purposes only. Data is represented as the total number of mice with statistical significance determined using a C [file supplementary_table_1.pdf]

Supplementary Table 1. Total number of *Men1*$^{+/+}$ *and Men1*$^{+/-}$ mouse births on C57BL/6 and 129S6/SvEv backgrounds, throughout the duration of the study, including mice generated for breeding purposes only. Data is represented as the total number of mice with statistical significance determined using a Chi-square test.

	Male			Female		
**Strain**	***Men1***$^{+/+}$	***Men1***$^{+/-}$		***Men1***$^{+/+}$	***Men1***$^{+/-}$	
**C57BL/6**	241	277	ns	325	349	ns
**129S6/SvEv**	271	253	ns	329	313	ns
