# Supplementary material for: Genetic background influences tumour development in heterozygous Men1 knockout mice
Source: Endocr Connect. 2020 Apr 28;9(5):426–37. doi: 10.1530/EC-20-0103 (PMC7274560; doi:10.1530/EC-20-0103)
Supplement: Supplementary Table 2. Survival of Men1+/+ and Men1+/- mice on C57BL/6 and 129S6/SvEv backgrounds between 18-27 months of age. Data is represented as a percentage, with the number of mice in brackets. Statistical significance was determined using a Fisher’s exact test (two tailed). [file supplementary_table_2.pdf]

Supplementary Table 2. Survival of *Men1*$^{+/+}$ *and Men1*$^{+/-}$ mice on C57BL/6 and 129S6/SvEv backgrounds between 18-27 months of age. Data is represented as a percentage, with the number of mice in brackets. Statistical significance was determined using a Fisher's exact test (two tailed).

Men1$^{+/+}$						***Men1***$^{+/-}$					
**Female**			**Male**			**Female**			**Male**		
**C56BL/6**	**129S6/SvEv**		**C56BL/6**	**129S6/SvEv**		**C56BL/6**	**129S6/SvEv**		**C56BL/6**	**129S6/SvEv**	
92.4 (66)	96.2 (79)	ns	100 (29)	97.0 (67)	ns	93.2 (89)	88.2 (76)	ns	91.7 (60)	79.7 (59)	ns

ns = not significant; +/+ = wild type; +/- = heterozygous.
