# Supplementary material for: Genetic background influences tumour development in heterozygous Men1 knockout mice
Source: Endocr Connect. 2020 Apr 28;9(5):426–37. doi: 10.1530/EC-20-0103 (PMC7274560; doi:10.1530/EC-20-0103)
Supplement: Supplementary Table 3. Variants identified in genes associated with tumourigenic pathways. [file supplementary_table_3.pdf]

**Supplementary Table 3.** Variants identified in genes associated with tumourigenic pathways.

| Gene | Ensembl_mouse_id | Chrom | Position | Reference | Variant | Functional Class |
|---|---|---|---|---|---|---|
| ***CCNE2*** | ENSMUSG00000028212 | chr4 | 11193052 | G | A | splice_region_variant |
| ***KRAS*** | ENSMUSG00000030265 | chr6 | 145217363 | T | C | 3_prime_UTR_variant |
| | | chr6 | 145217632 | T | G | 3_prime_UTR_variant |
| | | chr6 | 145218405 | T | C | 3_prime_UTR_variant |
| | | chr6 | 145218518 | C | G | 3_prime_UTR_variant |
| | | chr6 | 145218530 | T | C | 3_prime_UTR_variant |
| | | chr6 | 145218597 | T | G | 3_prime_UTR_variant |
| | | chr6 | 145218658 | C | G | 3_prime_UTR_variant |
| | | chr6 | 145218667 | A | G | 3_prime_UTR_variant |
| | | chr6 | 145218766 | T | C | 3_prime_UTR_variant |
| | | chr6 | 145218799 | C | T | 3_prime_UTR_variant |
| | | chr6 | 145218800 | C | T | 3_prime_UTR_variant |
| | | chr6 | 145219190 | C | T | 3_prime_UTR_variant |
| | | chr6 | 145219338 | G | A | 3_prime_UTR_variant |
| | | chr6 | 145219563 | G | C | 3_prime_UTR_variant |
| | | chr6 | 145219654 | C | T | 3_prime_UTR_variant |
| | | chr6 | 145220093 | C | G | 3_prime_UTR_variant |
| | | chr6 | 145220160 | C | T | 3_prime_UTR_variant |
| | | chr6 | 145220208 | G | T | 3_prime_UTR_variant |
| | | chr6 | 145247245 | A | G | 5_prime_UTR_variant |
| | | chr6 | 145220862 | C | T | intron_variant |
| | | chr6 | 145221538 | T | C | intron_variant |
| | | chr6 | 145221858 | T | C | intron_variant |
| | | chr6 | 145221874 | T | C | intron_variant |
| | | chr6 | 145221878 | T | C | intron_variant |
| | | chr6 | 145222272 | A | G | intron_variant |
| | | chr6 | 145222392 | A | G | intron_variant |
| | | chr6 | 145222414 | A | G | intron_variant |
| | | chr6 | 145222434 | A | G | intron_variant |
| | | chr6 | 145222453 | T | C | intron_variant |
| | | chr6 | 145222486 | A | C | intron_variant |
| | | chr6 | 145222502 | T | G | intron_variant |
| | | chr6 | 145222558 | T | C | intron_variant |
| | | chr6 | 145222894 | C | A | intron_variant |
| | | chr6 | 145223273 | G | A | intron_variant |
| | | chr6 | 145223402 | G | C | intron_variant |
| | | chr6 | 145224006 | C | A | intron_variant |
| | | chr6 | 145224868 | T | C | intron_variant |
| | | chr6 | 145225784 | C | G | intron_variant |
| | | chr6 | 145225876 | G | A | intron_variant |
| | | chr6 | 145225877 | G | C | intron_variant |
| | | chr6 | 145226029 | C | T | intron_variant |

| | | | | |
|---|---|---|---|---|
| chr6 | 145226068 | T | A | intron_variant |
| chr6 | 145226123 | G | A | intron_variant |
| chr6 | 145226727 | T | C | intron_variant |
| chr6 | 145226818 | A | G | intron_variant |
| chr6 | 145226841 | G | A | intron_variant |
| chr6 | 145226907 | G | A | intron_variant |
| chr6 | 145226946 | G | A | intron_variant |
| chr6 | 145227016 | G | A | intron_variant |
| chr6 | 145227152 | G | A | intron_variant |
| chr6 | 145227442 | T | C | intron_variant |
| chr6 | 145227482 | T | C | intron_variant |
| chr6 | 145227522 | A | G | intron_variant |
| chr6 | 145228062 | T | C | intron_variant |
| chr6 | 145228402 | G | A | intron_variant |
| chr6 | 145229922 | A | G | intron_variant |
| chr6 | 145230899 | T | C | intron_variant |
| chr6 | 145230909 | T | C | intron_variant |
| chr6 | 145231031 | T | C | intron_variant |
| chr6 | 145231310 | C | T | intron_variant |
| chr6 | 145231349 | G | A | intron_variant |
| chr6 | 145231362 | A | T | intron_variant |
| chr6 | 145231608 | A | T | intron_variant |
| chr6 | 145231904 | A | G | intron_variant |
| chr6 | 145231974 | T | A | intron_variant |
| chr6 | 145232693 | T | C | intron_variant |
| chr6 | 145232989 | A | G | intron_variant |
| chr6 | 145233009 | A | G | intron_variant |
| chr6 | 145233119 | G | A | intron_variant |
| chr6 | 145233521 | C | T | intron_variant |
| chr6 | 145233535 | T | G | intron_variant |
| chr6 | 145233546 | C | T | intron_variant |
| chr6 | 145233911 | A | G | intron_variant |
| chr6 | 145234202 | C | T | intron_variant |
| chr6 | 145234925 | G | C | intron_variant |
| chr6 | 145235339 | T | A | intron_variant |
| chr6 | 145236266 | C | T | intron_variant |
| chr6 | 145237530 | C | A | intron_variant |
| chr6 | 145238667 | A | T | intron_variant |
| chr6 | 145239032 | A | G | intron_variant |
| chr6 | 145239544 | A | G | intron_variant |
| chr6 | 145240174 | T | A | intron_variant |
| chr6 | 145241031 | A | T | intron_variant |
| chr6 | 145241200 | A | T | intron_variant |
| chr6 | 145241892 | C | T | intron_variant |
| chr6 | 145241929 | A | T | intron_variant |

| | | | | |
|---|---|---|---|---|
| chr6 | 145242096 | T | C | intron_variant |
| chr6 | 145242591 | G | A | intron_variant |
| chr6 | 145242955 | A | G | intron_variant |
| chr6 | 145242988 | A | T | intron_variant |
| chr6 | 145243597 | C | G | intron_variant |
| chr6 | 145243628 | T | C | intron_variant |
| chr6 | 145243673 | T | G | intron_variant |
| chr6 | 145243702 | T | G | intron_variant |
| chr6 | 145243705 | A | G | intron_variant |
| chr6 | 145243740 | A | G | intron_variant |
| chr6 | 145243852 | C | T | intron_variant |
| chr6 | 145243933 | G | T | intron_variant |
| chr6 | 145244594 | T | A | intron_variant |
| chr6 | 145245183 | T | C | intron_variant |
| chr6 | 145245441 | A | C | intron_variant |
| chr6 | 145245445 | A | C | intron_variant |
| chr6 | 145245449 | A | C | intron_variant |
| chr6 | 145245852 | T | C | intron_variant |
| chr6 | 145245900 | T | C | intron_variant |
| chr6 | 145245949 | G | A | intron_variant |
| chr6 | 145245970 | G | T | intron_variant |
| chr6 | 145245972 | C | T | intron_variant |
| chr6 | 145245974 | T | C | intron_variant |
| chr6 | 145245980 | G | A | intron_variant |
| chr6 | 145245982 | T | A | intron_variant |
| chr6 | 145245983 | G | A | intron_variant |
| chr6 | 145246313 | A | G | intron_variant |
| chr6 | 145246451 | C | T | intron_variant |
| chr6 | 145246462 | A | G | intron_variant |
| chr6 | 145246474 | G | T | intron_variant |
| chr6 | 145246511 | G | T | intron_variant |
| chr6 | 145246519 | C | G | intron_variant |
| chr6 | 145246916 | A | G | intron_variant |
| chr6 | 145246998 | A | G | intron_variant |
| chr6 | 145247714 | T | A | intron_variant |
| chr6 | 145247720 | C | T | intron_variant |
| chr6 | 145247819 | G | A | intron_variant |
| chr6 | 145247833 | T | G | intron_variant |
| chr6 | 145247894 | G | T | intron_variant |
| chr6 | 145248251 | A | G | intron_variant |
| chr6 | 145248332 | C | T | intron_variant |
| chr6 | 145248963 | T | C | intron_variant |
| chr6 | 145249342 | A | G | intron_variant |
| chr6 | 145249877 | C | G | intron_variant |
| chr6 | 145250371 | C | T | upstream_gene_variant |

| | | | | | | |
|---|---|---|---|---|---|---|
| | | chr6 | 145252497 | G | A | upstream_gene_variant |
| | | chr6 | 145253331 | G | A | upstream_gene_variant |
| | | chr6 | 145253338 | A | T | upstream_gene_variant |
| | | chr6 | 145253401 | G | T | upstream_gene_variant |
| | | chr6 | 145253511 | A | C | upstream_gene_variant |
| | | chr6 | 145253517 | G | A | upstream_gene_variant |
| | | chr6 | 145253554 | G | A | upstream_gene_variant |
| | | chr6 | 145253827 | A | C | upstream_gene_variant |
| | | chr6 | 145253843 | A | G | upstream_gene_variant |
| | | chr6 | 145254574 | T | G | upstream_gene_variant |
| | | chr6 | 145254949 | G | A | upstream_gene_variant |
| ***WNT2B*** | ENSMUSG00000027840 | chr3 | 104945409 | A | C | 3_prime_UTR_variant |
| | | chr3 | 104945476 | T | C | 3_prime_UTR_variant |
| | | chr3 | 104946432 | G | A | 3_prime_UTR_variant |
| | | chr3 | 104946891 | A | G | 3_prime_UTR_variant |
| ***IL3RA*** | ENSMUSG00000068758 | chr14 | 14351982 | G | A | missense_variant |
| | | chr14 | 14349243 | A | G | missense_variant |
| | | chr14 | 14351935 | T | C | missense_variant |
| | | chr14 | 14349324 | C | T | missense_variant |
| | | chr14 | 14350757 | G | C | missense_variant |
| | | chr14 | 14349332 | A | G | missense_variant |
| | | chr14 | 14348817 | G | A | splice_region_variant |
| | | chr14 | 14349379 | G | T | splice_region_variant |
| | | chr14 | 14351170 | T | C | splice_region_variant |
| | | chr14 | 14346499 | T | C | 5_prime_UTR_variant |
| | | chr14 | 14346503 | C | T | 5_prime_UTR_variant |
| | | chr14 | 14347024 | G | T | 5_prime_UTR_variant |
| | | chr14 | 14347112 | C | A | 5_prime_UTR_variant |
| | | chr14 | 14347113 | C | A | 5_prime_UTR_variant |
| | | chr14 | 14347114 | A | G | 5_prime_UTR_variant |
| | | chr14 | 14355476 | G | A | 3_prime_UTR_variant |
| ***TNFRSF10A*** | ENSMUSG00000022074 | chr14 | 69775097 | G | A | missense_variant |
| | | chr14 | 69773437 | A | G | missense_variant |
| | | chr14 | 69773438 | T | G | missense_variant |
| | | chr14 | 69776162 | G | C | missense_variant |
| | | chr14 | 69782337 | A | G | missense_variant |
| | | chr14 | 69777760 | C | T | missense_variant |
| | | chr14 | 69767783 | G | A | missense_variant |
| | | chr14 | 69775091 | G | T | missense_variant |
| | | chr14 | 69775128 | T | A | missense_variant |
| | | chr14 | 69767477 | T | G | 5_prime_UTR_variant |
| | | chr14 | 69767481 | C | T | 5_prime_UTR_variant |
| | | chr14 | 69782770 | T | G | 3_prime_UTR_variant |
| | | chr14 | 69783279 | G | A | 3_prime_UTR_variant |
| ***COQ7*** | ENSMUSG00000030652 | chr7 | 118509955 | G | A | missense_variant |

| | | | | | | |
|---|---|---|---|---|---|---|
| | | chr7 | 118525817 | C | G | splice_region_variant |
| | | chr7 | 118510146 | C | T | stop_gained |
| | | chr7 | 118525104 | C | T | 3_prime_UTR_variant |
| | | chr7 | 118525149 | T | A | 3_prime_UTR_variant |
| | | chr7 | 118525163 | T | C | 3_prime_UTR_variant |
| ***DMPK*** | ENSMUSG00000030409 | chr7 | 19093085 | G | A | missense_variant |
| | | chr7 | 19092453 | T | G | stop_gained |
| | | chr7 | 19093647 | C | T | 3_prime_UTR_variant |
