# Supplementary material for: Genetic background influences tumour development in heterozygous Men1 knockout mice
Source: Endocr Connect. 2020 Apr 28;9(5):426–37. doi: 10.1530/EC-20-0103 (PMC7274560; doi:10.1530/EC-20-0103)
Supplement: Supplementary Table 4. Multiple endocrine neoplasia type I (MEN1) conventional mouse knockout models and their background strains. [file supplementary_table_4.pdf]

Supplementary Table 4. Multiple endocrine neoplasia type I (MEN1) conventional mouse knockout models and their background strains.

Model	1	2	4	5
Type[a]				
Men1 exons deleted	3-8	3	2	1-2
Replacement cassette[b]	PGK-Neo	Neo-TK	PGK-Neo	PGK-Neo
Strain[c]	Mixed NIH Black Swiss/129	129	Mixed C57/129	Mixed C57/129
Tumour development[d]				
Parathyroid	+	+	+	+ (↑Ca)
Pancreas	+ (INS)	+ (INS, GLU)	+ (INS, GLU)	+ (INS,GLU,)
Pituitary	+ (PRL)	+ (PRL, GH)	+ (PRL, NF)	+ (PRL, GH, ACTH)
Adrenal cortex	+	+	+	+ (↑Cort)
Phaeochromocytoma	+	-	+	+
Gastric NET	+	-	-	-
Thyroid	+	+	+	+
Testicular	-	+ (LD)	+ (LD)	+ (LD)
Extra pancreatic gastrinoma	-	+	-	-
Ovarian	-	+ (SC)	+ (SC)	+ (SC)
References	(Crabtree, *et al.* 2001)	(Bertolino, *et al.* 2003)	(Loffler, *et al.* 2007)	(Harding, *et al.* 2009)

[a]PKG-Neo, phosphoglycerate kinase-neomycin; Neo-TK, neomycin-thymidine kinase
[b]129-129S6/SvEv; C57-C57BL/6
[c]↑Ca, hypercalcaemia; INS, insulin; GLU, glucagon; PRL, prolactin; GH, growth hormone; NF, non-functioning; ACTH, adrenocorticotrophin; NET, neuroendocrine tumours; LD, Leydig cell tumour; SC, sex cord stromal cell tumours; Cort, corticosterone
